# Unveiling bulk and surface radiation forces in a dielectric liquid

**DOI:** 10.1038/s41377-022-00788-7

**Published:** 2022-04-20

**Authors:** N. G. C. Astrath, G. A. S. Flizikowski, B. Anghinoni, L. C. Malacarne, M. L. Baesso, T. Požar, M. Partanen, I. Brevik, D. Razansky, S. E. Bialkowski

**Affiliations:** 1 Department of Physics, Universidade Estadual de Maringá, Maringá, PR Brazil; 2 Faculty of Mechanical Engineering, University of Ljubljana, Ljubljana, Slovenia; 3 Department of Electronics and Nanoengineering, Aalto University, Aalto, Finland; 4 Department of Energy and Process Engineering, Norwegian University of Science and Technology, Trondheim, Norway; 5 Institute for Biomedical Engineering and Institute of Pharmacology and Toxicology, Faculty of Medicine, University of Zurich, Zurich, Switzerland; 6 Institute for Biomedical Engineering, Department of Information Technology and Electrical Engineering, ETH Zurich, Zurich, Switzerland; 7 Department of Chemistry and Biochemistry, Utah State University, Logan, UT USA

**Keywords:** Optical physics, Optical physics, Optical techniques

## Abstract

Precise control over light-matter interactions is critical for many optical manipulation and material characterization methodologies, further playing a paramount role in a host of nanotechnology applications. Nonetheless, the fundamental aspects of interactions between electromagnetic fields and matter have yet to be established unequivocally in terms of an electromagnetic momentum density. Here, we use tightly focused pulsed laser beams to detect bulk and boundary optical forces in a dielectric fluid. From the optical convoluted signal, we decouple thermal and nonlinear optical effects from the radiation forces using a theoretical interpretation based on the Microscopic Ampère force density. It is shown, for the first time, that the time-dependent pressure distribution within the fluid chiefly originates from the electrostriction effects. Our results shed light on the contribution of optical forces to the surface displacements observed at the dielectric air-water interfaces, thus shedding light on the long-standing controversy surrounding the basic definition of electromagnetic momentum density in matter.

## Abstract (web summary)

Laser pulses generate pressure waves due to electrostriction effects decoupling bulk and boundary optical forces in water, probed by the convoluted optical signal due to the resulting spatiotemporal pressure distribution.

## Graphical abstract

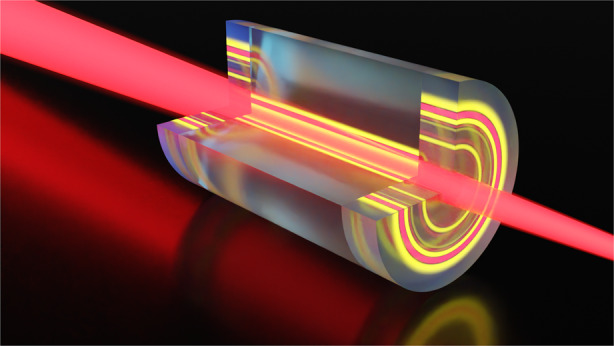

## Introduction

Electromagnetic fields store linear momentum, accounting for the radiation pressure resulting from light-matter interactions responsible for many important nanotechnology applications^1–5^. Predicted by Maxwell in 1873^6^, the fundamental aspects of radiation pressure physics still remain uncertain in regard to the correct definition of an electromagnetic momentum density in a medium^7–11^. First proposed by Minkowski in 1908^12^ and followed by Abraham in 1909^13^, the ongoing discussion on the form taken by the momentum of light in matter, the classic Abraham–Minkowski controversy, has been extensively described in many notable theoretical and experimental contributions^14–32^.

The problem associated with this controversy appears to be a simple choice of the most suitable expression for the electromagnetic momentum density in matter. The implications of such a choice are however far reaching, being driven by many of the most precise experimental tools available today. To illustrate the origin of this fascinating problem, the energy-momentum tensor proposed by Minkowski predicts a momentum proportional to the refractive index of the medium, *n*, and to the momentum in vacuum, *p*_0_, as $p_{{{\mathrm{M}}}} = np_0$, while Abraham predicts a momentum $p_{{{\mathrm{A}}}} = p_0/n$. It turns out that both energy-momentum tensors lead to common electromagnetic forces at interfaces that can be equally used to describe most of the experimental results obtained to date.

Theoretically, the controversy has been resolved by identifying the kinetic momentum via canonical momentum proposed by Abraham and Minkowski’s description^17^. The canonical momentum is the total momentum of light, while the kinetic momentum is the electromagnetic momentum when the rest of the total momentum is deposited locally in the material by a force density term called the Abraham force. This would settle the debate and present both momenta as legitimate yet describing different aspects of the electromagnetic wave phenomena^14,17,33^. Although this assumption may sound appealing, some experimental results are not fully contemplated by this approach. Considering the limited experimental evidence collected thus far^16,17^, the controversy continues to this day, despite many distinguished authors claim to have settled the debate.

On the experimental side, many of the fairly important experiments on radiation optics to date rely on detecting the effects of electromagnetic forces at the dielectric boundaries produced by the interaction between light and matter. Would it be different if the local bulk optical forces, in addition to the surface effects, could be detected with the same experimental configuration? May such experiments help to decouple volume and boundary forces? The answer to these questions should incorporate all the time-dependent optical forces acting within matter, retaining the characteristics of radiation pressure effects observed at the dielectric surface. Nonlinear effects should be carefully considered in the description.

Here, we use an all-optical pump and probe lensing technique to detect the electromagnetic forces induced by a pulsed laser beam tightly focused into water. The effects of radiation forces are decoupled from the optical Kerr and thermal effects induced in the liquid. The local effect caused by the rapidly varying electrostriction force is measured directly, and a theoretical interpretation is presented to explain in detail all the phenomena observed at the interface between air and water.

## Experimental set-up

**Fig. 1:**
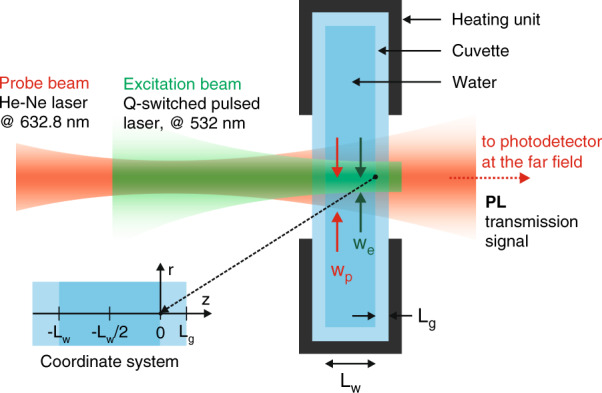
Photo-induced lensing (PIL) setup. Schematic of the time-dependent photo-induced lensing measurement set-up. Green and red routes represent pump and probe laser beams, respectively. The temperature was fixed at (298.15 ± 0.08) K. A complete experimental description is presented in the Methods section

To experimentally detect the dynamics of the pressure-induced acoustic waves by optical forces within water, we exploit a highly sensitive detection of wavefront distortions by a time-dependent photo-induced lensing (PIL) technique (Fig. 1). Nanosecond laser pulses irradiate the sample, changing the local pressure due to the radiation forces in addition to a small heat deposition. Nonlinear optical Kerr effect is also observed during the pulse duration. A low-irradiance laser beam traverses the sample thus probing the induced effects. The intensity of the probe beam is monitored in the far field by a fast photodetector (see Methods).

**Fig. 2:**
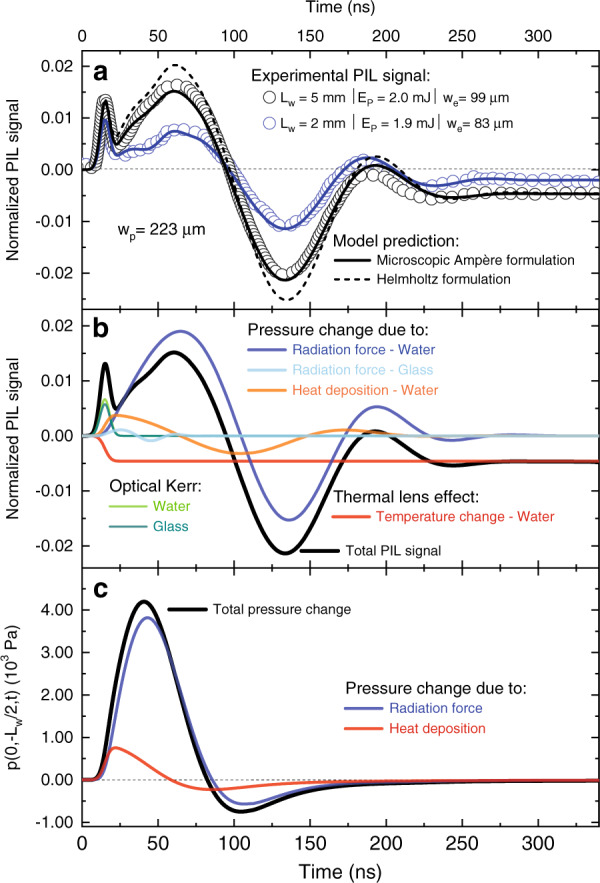
Time-dependent photo-induced lensing (PIL) transients. **a** PIL signal under pulsed laser excitation at 532 nm. The transients show the intensity variation of the centre of a continuous probe laser beam transmitted through the cuvette-water interfaces measured by a photodetector in the far-field. Open symbols are experimental data and continuous lines represent the numerical calculations using *S*(*t*); confidence level of 95%. The uncertainties in **a** are smaller than 1% and correspond to the standard deviation of the mean over all the experiments (see Methods). The optical path length of the cuvette was *L*_w_ (2 mm and 5 mm) with the cuvette walls having a thickness of *L*_g_ = 1.25 mm. **b** Numerical calculations of the individual optical forces in the water and in the quartz cuvette walls showing the radiation forces along with contributions of the thermal and optical Kerr effects to the PIL intensity signal. **c** Pressure calculated at the centre of the water sample with contributions due to radiation forces and thermal deposition

The probe beam wavefront distortion caused by the radiation forces are detected both in the water (Milli-Q water) and in the quartz cuvette walls (fused silica), on both ends. The results for the photo-induced lensing signals are presented in Fig. 2a (open symbols). The transients show the intensity variation in the centre of the probe beam transmitted through the cuvette-water sample, as measured by the photodetector. The probe beam intensity increases within short time due to a focusing effect taking place during the laser pulse duration (Fig. 2a for *L*_w_ = 2.0 mm and *L*_w_ = 5.0 mm). This effect is caused by a fast nonlinear effect altering the refractive index of the sample with the pulse intensity. Subsequently, the signal follows an oscillatory behaviour towards a steady-state, exhibiting a combination of several transient effects occurring in the sample. Note that the amplitude of the steady-state signal is below the initial signal, which accounts for the slower process of heat diffusion.

The wavefront distortion sensed by the probe beam originates from the non-uniform excitation interaction with the sample leading to an increase in the internal energy, the latter being dispersed in two different modes of hydrodynamic relaxation. The increased internal energy results in a temperature change *T*(*r*, *z*, *t*) in the sample or the coupling material placed next to the sample. This temperature change results in a change in sample density. If the photothermally induced temperature alteration occurs faster than the time required for the fluid to expand (or contract, in some cases), then the rapid temperature change will result in a pressure change *p*(*r*, *z*, *t*). The pressure perturbation then relaxes by emitting an acoustic wave. Once the pressure has relaxed to its equilibrium level, a density change proportional to the temperature will remain. The time-dependent intensity signal detected in the experiments shows only the centre of the probe beam spot at the detector plane in the far-field region. The calculation of the PIL signal requires the determination of the temperature and pressure fields considering all the effects of the radiation forces in the liquid and in the cuvette walls (see Methods).

## Theory

In a fluid, electrostriction plays a significant role in establishing local pressure, even though it does not contribute to the total force on a dielectric body^24^. The general force density includes electrostriction and magnetostriction terms derived by solely considering static fields. Its use for time-dependent fields relies on phenomenological approaches. The force identifies the momentum density as *G* = *E* × *H*/*c*^2^ and, assuming a dielectric non-magnetic fluid, the force density is given by^13,14,34^

1 $$\begin{array}{*{20}{ll}} {f = - {\displaystyle\frac{1}{2}}\varepsilon _0|{{{\mathbf{E}}}}|^2\nabla \varepsilon _{{{\mathrm{r}}}} + {\displaystyle\frac{1}{2}}\varepsilon _0\nabla \left[ {\rho _{{{\mathrm{m}}}}{\displaystyle\left( {\frac{{\partial \varepsilon _{{{\mathrm{r}}}}}}{{\partial \rho _{{{\mathrm{m}}}}}}} \right)}_T|{{{\mathbf{E}}}}|^2} \right]} \\\qquad { + {\displaystyle\frac{{\varepsilon _{{{\mathrm{r}}}} - 1}}{{c^2}}}{\displaystyle\frac{\partial }{{\partial t}}}\left( {\mathbf{E} \times \mathbf{H}} \right)} \end{array}$$

where *ρ*_m_ is the mass density, *c* is the speed of light in vacuum, *E* and *H* represent the electric and magnetic fields, *ε*_0_ is the permittivity in vacuum and $\varepsilon _{{{\mathrm{r}}}} = \varepsilon /\varepsilon _0$ is medium’s relative permittivity. The first term in Eq. (1) is a common term arising from the Minkowski and Abraham energy-momentum tensors and is often called the Abraham-Minkowski force (**f**^AM^) acting where relative permittivity exhibits spatial variations, especially along interfaces where *ε* presents a discontinuity. The second term is the electrostriction force, which becomes important when the field and dielectric permittivity are inhomogeneous. The existence of this term was demonstrated in experiment with quasi-stationary fields^26^. The last term is known as the Abraham force term (**f**^Ab^). It averages to zero at optical frequencies and can be neglected in our model. In the absence of Abraham force term, Eq. (1) reduces to the Helmholtz force^34^.

For a laser beam normally incident from free space (air) into a dielectric liquid (inside a cuvette), the electrostriction force acts within the dielectric fluid and in the glass walls towards higher field strength, causing a local pressure increase in the centre of a laser beam propagating through the media. From Eq. (1), the time averaged Helmholtz volume force <*f*_H_> is written as

2 $$\langle f_{{{\mathrm{H}}}}\rangle = \frac{1}{2}\varepsilon _0\nabla _r\left[ {\rho _{{{\mathrm{m}}}}\left( {\frac{{\partial \varepsilon _{{{\mathrm{r}}}}}}{{\partial \rho _{{{\mathrm{m}}}}}}} \right)_T{\langle{E_{||}}}\rangle^2} \right]$$

Here, $\left\langle {E_{||}^2} \right\rangle = [4/(n + 1)^2]\left\langle {E_{{{{\mathrm{inc}}}}}^2} \right\rangle$ is the electric field tangential to the water surface and $\left\langle {E_{{{{\mathrm{inc}}}}}^2} \right\rangle$ is the incident electric field. The field intensity is $I\left( {r,t} \right) = \varepsilon _0c\left\langle {E_{{{{\mathrm{inc}}}}}^2} \right\rangle$. The Gaussian pulsed laser beam intensity is described by $I(r,t) = (2E_{{{\mathrm{P}}}}/t_0\pi w_{{{\mathrm{e}}}}^2){{{\mathrm{exp}}}}\left( { - 2r^2/w_{{{\mathrm{e}}}}^2} \right){{{\mathrm{exp}}}}\left[ { - (t - \xi )^2/\tau ^2} \right]$, where the liquid is assumed to obey the Clausius-Mossotti relation, $\rho _{{{\mathrm{m}}}}\left( {\partial \varepsilon _{{{\mathrm{r}}}}/\partial \rho _{{{\mathrm{m}}}}} \right)_T = (\varepsilon _{{{\mathrm{r}}}} - 1)(\varepsilon _{{{\mathrm{r}}}} + 2)/3$.

On the other hand, the conventional Ampère formulation (also known as Lorentz formulation) does employ the correct microscopic model with respect to bound charges and currents, but those are not expected to correctly depict the microscopic force distribution inside matter^35^. Thus, it seems plausible to consider a formulation that arises directly from the charges and current distributions related to microscopic electric and magnetic dipoles. In particular, the Microscopic Ampère formulation considers an electromagnetic force density acting on a dielectric medium with no free charges or current, as elaborated in Supplementary Information. This force density is implicitly analysed at distances where only the dipolar contribution of the source is assumed to be relevant. Mathematically, the electric charge and current densities are given by $\rho ({{{\mathbf{r}}}},t) = - ({{{\mathbf{p}}}} \cdot \nabla )\delta ^3({{{\mathbf{r}}}})$ and ${{{\mathbf{J}}}}({{{\mathbf{r}}}},t) = {{{\dot{\mathbf p}}}}\delta ^3({{{\mathbf{r}}}}) - ({{{\mathbf{m}}}} \times \nabla )\delta ^3({{{\mathbf{r}}}})$^35^, where **p** and **m** are the dipole’s electric and magnetic moment, respectively. The force acting on the dielectric is given by the continuous version of the experimental Lorentz force law, ${{{\mathbf{F}}}} = {\int} {\left( {\rho {{{\mathbf{E}}}} \,+ \,{{{\mathbf{J}}}} \,\times\, {{{\mathbf{B}}}}} \right){{{\mathrm{d}}}}^3{{{\mathbf{r}}}}}$, leading to ${{{\mathbf{f}}}}_{{{{\mathrm{MA}}}}} = \left( {{{{\mathbf{P}}}} \cdot \nabla } \right){{{\mathbf{E}}}} + {{{\dot{\mathbf P}}}}\, \times\, {{{\mathbf{B}}}} + {{{\mathbf{M}}}} \times \left( {\nabla \times {{{\mathbf{B}}}}} \right) + \left( {{{{\mathbf{M}}}} \cdot \nabla } \right){{{\mathbf{B}}}}$. Using Maxwell’s equations, and considering a linear isotropic media, in which the medium responses are given by ${{{\mathbf{P}}}} = \varepsilon _0\chi _{{{\mathrm{e}}}}{{{\mathbf{E}}}}$ and ${{{\mathbf{M}}}} = \chi _{{{\mathrm{m}}}}{{{\mathbf{H}}}}$, and the hidden momentum contribution, the force density can be written as

3 $$\begin{array}{*{20}{c}} {{{{\mathbf{f}}}}_{{{{\mathrm{MA}}}}} = {\displaystyle\frac{1}{2}}\nabla \left( {{{{\mathbf{P}}}} \cdot {{{\mathbf{E}}}}} \right) + {\displaystyle\frac{1}{2}}\nabla \left( {{{{\mathbf{M}}}} \cdot {{{\mathbf{B}}}}} \right) - {\displaystyle\frac{1}{2}}|{{{\mathbf{E}}}}|^2\nabla \varepsilon } \\ { - {\displaystyle\frac{1}{2}}|{{{\mathbf{H}}}}|^2\nabla \mu + {\displaystyle\frac{{n^2 - 1}}{{c^2}}}{\displaystyle\frac{\partial }{{\partial t}}}\left( {{{{\mathbf{E}}}} \times {{{\mathbf{H}}}}} \right)} \end{array}$$

which is valid for linear, isotropic inhomogeneous media. The presence of free sources would generate the extra terms *ρ***E** and **J** × **B**, and can be included if necessary. One can identify the first and second terms as the electrostriction and magnetostriction force densities, respectively. The third and fourth terms are the usual Abraham-Minkowski force density, while the last term is the Abraham force density. This representation arguably contemplates nearly every aspect of the reported experiments, and arises naturally from an explicit and simple microscopic model, with no need for phenomenological approaches. Note that for a non-magnetic, linear isotropic dielectric medium, the Microscopic Ampère force density reduces to the well-known Einstein-Laub force density^36^. As shown in Methods, for optical fields in non-magnetic media, it also becomes equal to the force density in Eq. (1). The time-average over an optical cycle for the Microscopic Ampère formulation yields

4 $$\langle f_{{{{\mathrm{MA}}}}}\rangle = \frac{2}{c}\frac{{(n - 1)}}{{(n + 1)}}\nabla _rI(r,t)$$

which, as compared to the Helmholtz approach in Eq. (2), attains a relative magnitude for the volume forces of $\langle f_{{{{\mathrm{MA}}}}}\rangle /\langle f_{{{\mathrm{H}}}}\rangle \approx 0.8$ for water (*n* = 1.33). This relatively small difference between the Microscopic Ampère force and the phenomenological electrostriction force, as given by the Helmholtz approach, can readily be detected as outlined in the following. It ought to be mentioned that these expressions have been tested for fluids in the classic electrostriction experiment of Hakim and Higham^37^ by measuring refractive index increase. Although the results agreed with the Helmholtz equation (within 5%), the experiments were performed in non-polar fluids exposed to a strong electric field.

The effects of the radiation forces in the liquid and in the cuvette walls can be calculated by solving the heat diffusion equation in addition to the non-homogeneous acoustic wave equations with appropriate boundary conditions. The thermal effects caused by laser absorption in the liquid are small due to its very low optical absorption coefficient. We applied the finite element analysis method for the numerical calculations and used Comsol Multiphysics software (COMSOL Inc, Burlington, MA, USA) to solve these equations. The time-dependent solutions for the pressure *p*(*r*, *z*, *t*) and temperature *T*(*r*, *z*, *t*) are retrieved from the models (see Methods) and used to calculate the phase shifts and PIL intensity signals.

## Results

**Fig. 3:**
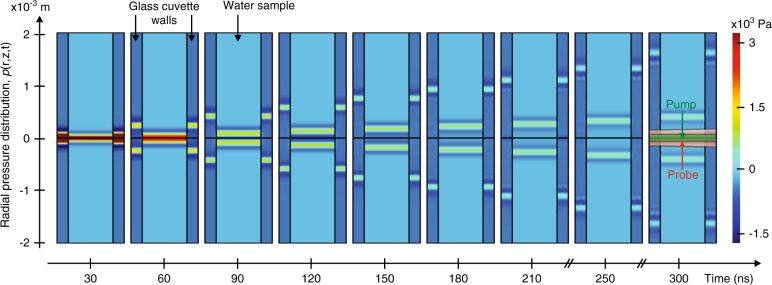
Radial pressure distribution. Time evolution of the pressure distribution in the water and cuvette walls under pulsed excitation. *p*(*r*, *z*, *t*) was calculated using the parameters listed in Supplementary Table S2

Figure 2a shows the calculated PIL signals (lines) using the Microscopic Ampère formulation in Eq. (4). Note that the numerical predictions (continuous lines) are in excellent agreement with the experimental data. Residuals are shown in Supplementary Fig. S1. Figure 2b, c shows the contributions from each effect to the PIL signal and the pressure changes over time at the centre of the water column. The complex form of the acoustic waves dispersed in the water and cuvette walls during laser excitation can be calculated using 2D simulation in Comsol. Figure 3 displays the actual pressure distribution in the water-cuvette sample at different times. The parameters used for the calculations are presented in Supplementary Table S2. The normalized PIL signal is calculated using the probe beam intensity signal *S*(*t*) by taking into account the different phase shifts induced by optical Kerr, thermal and radiation force contributions (see Methods).

Under high energy density deposition conditions, such as used in these experiments, the refractive index of the samples maintain strong dependence on the optical intensity. This intensity-dependent refractive index or nonlinear index of refraction, *n*_2_, gives the rate at which the index changes with the laser intensity, which is known as the optical Kerr effect^38^. As both water and fused silica have *n*_2_ of the same magnitude (see Supplementary Table S2), the first peak observed in the PIL signal, which follows the time dependence of the laser pulse, is a direct manifestation of the phase shift induced on the probe beam by the optical Kerr effect. Note that this effect contributes to the signal only during the laser pulse (Fig. 2b).

The optical absorption coefficient of water at 532 nm is approximately 0.045 m^−1^. Although very small, it causes a temperature increase of a few mK that, combined with the large ∂*n*/∂*T*, generates a transient thermal lens effect, as shown in Fig. 2b (red line). The optical absorption coefficient of fused silica is even smaller, by two orders of magnitude, hence thermal lensing in the walls can be safely neglected. The behaviour of the thermal lens effect dictates the PIL steady state signal.

The larger contribution to the PIL signal comes from the pressure waves propagating laterally from the excitation volume within the samples, both caused by the radiation force (electrostriction) and by heat-deposition-induced pressure gradient. The effect of radiation forces was only perceived in the experiments due to a fine combination of thermal, optical, and acoustic properties of the samples. The electrostrictive force introduces the effect of compressibility in the liquid, and one expects the time required for pressure equilibrium to be established in the water should be of the same order as the time needed for sound to traverse the probed volume (with radius *w*_p_). Due to the finite excitation beam width *w*_e_, the acoustic waves require (*w*_p_ + *w*_e_)/*v* ≈ 220 ns to travel such a distance, in congruence with the experimental results (Fig. 2). The argument used here also agrees well also with Brevik’s analysis of the Ashkin-Dziedzic experiment from 1973^25^, observing the outward bulge of the free surface of water illuminated by a pulsed laser^15^.

The curves in blue and orange in Fig. 2b are the pressure contributions from radiation forces and heat deposition to the PIL signal, respectively. Note that, even though being smaller in magnitude, thermal waves still shape the final form of the transients. As the temperature change in the walls is negligible, thermal waves do not contribute to the signal generated in the walls, while the optical pressure produces a very weak signal (light blue line). This minor effect has also been observed experimentally in a bulk sample of fused silica measured using the same PIL experimental configuration (not shown here). The overall PIL signal consists of an integrated contribution by all these distinct effects occurring simultaneously in the sample, hence representing radiation forces interacting with matter. All the forces, in addition to the optical Kerr effect occurring during the pulse duration, represent the total experimental PIL signal observed in the experiments. All these effects are linearly dependent on the optical path length and excitation pulse energy (not shown here), and so are the experimental signals observed by changing the dimensions of the cuvette from 2 mm to 5 mm, as displayed in Fig. 2a. Evidently, the numerical calculations using the Microscopic Ampère formulation in Eq. (4) are in excellent agreement with our experimental results. On the other hand, the same calculation using the Helmholtz approach in Eq. (2) overestimates the experimental signal (dashed line, Fig. 2a).

## Discussion

The notion that electrostriction may play a non-negligible role in acoustic wave generation by transient illumination of weakly absorbing liquid phase was first introduced by the optoacoustic community in the late 1970’s^39–41^. Theoretical treatments of optoacoustic source generation via both thermal expansion and electrostriction simultaneously^39,42–45^ revealed two important times scales: the temporal duration of the excitation pulse ($\tau _{{{\mathrm{p}}}} \sim 10$ ns in the current work) and the acoustic transit time ($\tau _{{{\mathrm{a}}}} = w_{{{\mathrm{e}}}}/v \sim 68$ ns) across the excitation beam radius. When $\tau _{{{\mathrm{p}}}} < \tau _{{{\mathrm{a}}}}$, as in our experiments, the spatial extent of the pulse plays a more significant role than its temporal span. This in known as a wide (broad) beam case of a line (cylindrical) optoacoustic source^40,43,46^. The acoustic pressure *p* can be written as the sum of a thermal expansion term *p*_th_ and an electrostriction term *p*_el_ with the latter being proportional to the time derivative of *p*_th_. Hence, whenever *p*_th_ reaches its peak, *p*_el_ crosses the zero value^40,47,48^. This also appears to be true for the thermal and the electrostrictive parts of the PIL signal in Fig. 2b. The ratio between the pressure amplitudes in our case is $|p_{{{{\mathrm{el}}}}}/p_{{{{\mathrm{th}}}}}| \approx 6$, as derived by Heritier^44^. This pressure ratio also corresponds to the PIL signal ratio of the pressure wave components due to electrostriction and thermal expansion (see Fig. 2b). This is the first measurement where electrostriction has a dominant contribution to the final signal, thus enabling the elaborate investigation of the electrostriction effect, especially its magnitude, which depends on the electromagnetic formalism adopted. Suppression of electrostriction has been experimentally demonstrated by using suitable time-gated detection of the acoustic signal^40,49,50^. In solids, electrostrictive mixing of two laser beams may lead to observable effects, as previously noted by Cachier^51^. Electrostrictive counterforce on fluid microdroplet illuminated by a laser pulse shows that if the pulse is short in comparison with the transit time, the disruptive optical Abraham–Minkowski radiation force is countered by electrostriction, and the net stress is compressive. Long pulses cancel electrostriction by elastic pressure and the surviving term of the electromagnetic force, the Abraham–Minkowski force^52^.

The present work demonstrates that the system is robustly represented by our outlined model of radiative force transfer. The expression used for the bulk forces in the liquid and glass walls from the Microscopic Ampère force density is presumed to be the total force density acting in the samples. Most recently, measurements of the opto-mechanical forces generated by laser beams inside optical fibers have been performed by analysing the symmetry of the mechanical oscillations in the solid dielectric^53^. It has been shown that the measured force is consistent with the Helmholtz approach. As we know, the Helmholtz force is the same as the Abraham-Minkowski force (at stationary condition when the Abraham term is omitted), augmented by the electrostriction term. The results showed that the electromagnetic force neither resembles the Lorentz (conventional Ampère) nor Einstein-Laub forms, but a combination of both (see Supplementary Information for details on the different formulations). The Microscopic Ampère formulation can also be used to describe force density components related to spatial variations of the refractive index, which is known to occur in optical fibers. This effect, together with the ellipsoidal shape of the cross-section of the real fiber, could lead to the reported loss of azimuthal symmetry in the forces observed in ref. ^53^.

**Fig. 4:**
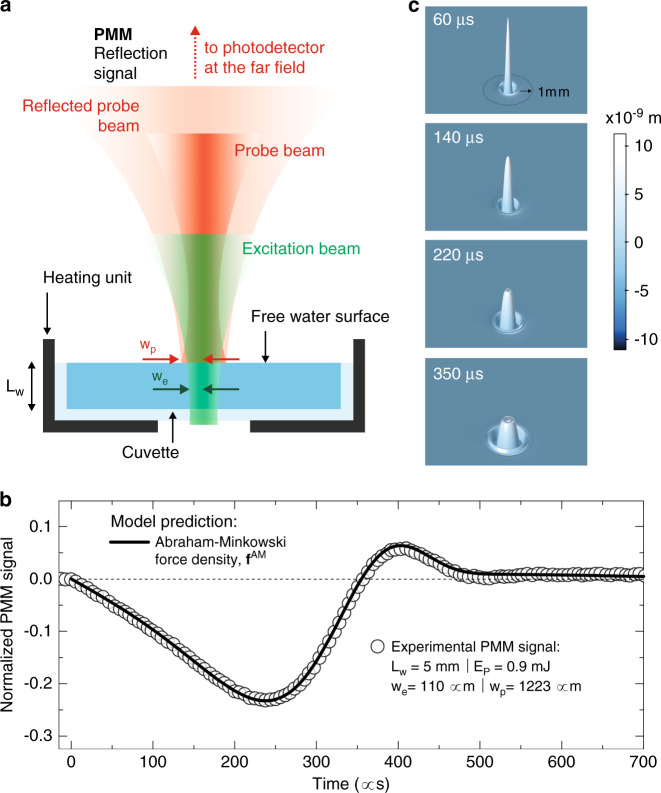
Photomechanical mirror (PMM). **a** Schematic diagram of the apparatus for the time-resolved PMM experiment. Details are given in ref. ^31^. **b** PMM signal under pulsed laser excitation at 532 nm. The transient shows the intensity variation of the centre of a continuous probe beam laser beam reflected off of the water surface measured at the photodetector. Open symbols are experimental data and continuous line represents the numerical calculations using Eq. (4). Dashed line shows the Helmholtz model prediction. **c** Time evolution of the water surface deformation

This recalls a captivating question regarding the form of the pressure exerted by this bulk force on a free water surface with perpendicular pulse excitation. Consider, for instance, the experiments performed in ref. ^31^, in which normally incident tightly focused laser pulses generated surface deformations at the air–water interface. It has been shown that the liquid surface experienced a net outward force. The quantitative analysis predicted that the overall pressure elevating the liquid surface can be expressed as $p\left( {r,t} \right) = - 2[(n - 1)/(n + 1)]I\left( {r,t} \right)/c$. We have conducted additional experiments to detect the surface displacement of a free water surface using an experimental setup with similar parameters as the PIL setup described in this work. The photomechanical mirror (PMM) setup was used to detect the surface deformation of water. Figure 4a–c shows the schematics of the experiments^31^ and the transient signal with the corresponding time evolution of the surface displacement of water not exceeding a few nanometres. The results are, in fact, in complete agreement with the Microscopic Ampère force density in Eq. (4) (Residuals are shown in Supplementary Fig. S2), as a bulk compressive electrostrictive force immediately arises after the pulse, producing elastic deformations propagating inwards, as described previously. At the free surface, additional electrostriction presses the surface downwards against the Abraham-Minkowski surface force density^15^. The perfect short-time balance between mutually cancelling bulk and surface electrostriction forces presents, undeniably, a solid evidence of the effect of Abraham-Minkowski force density on the surface displacement in addition to gravity and surface tension effects^31^. The continuous line in Fig. 4b shows the theoretical calculation of the signal using the Microscopic Ampère force density (continuous line). The prediction is in excellent agreement with the experiments. Note that, while the present work unambiguously resolves the radial component of the force density, measurement of the longitudinally acting Abraham force remains an open question that needs further investigations.

Even though substantial efforts have so far been devoted to addressing the question of the basic definition of electromagnetic momentum density in matter, this fundamental problem in classical and quantum mechanical theory of electrodynamics has not been unequivocally resolved. Our work thus represents a bold step in this direction, facilitating resolution of the century old Abraham-Minkowski controversy.

## Materials and methods

### Photo-induced lensing technique

The time-resolved photo-induced lensing method used in this work is illustrated in Fig. 1 and described in detail in ref. ^54^. A pulsed TEM_00_ laser operating at 532 nm (Q-switched diode-pumped laser, Innolas, Model Dry-S, custom made) with a FWHM pulse width of 9 ns was used to excite the sample. The laser beam was focused in the sample using a *f* = 0.50 m focal length lens. A low-irradiance TEM_00_ He-Ne laser at 632.8 nm (Thorlabs, Model 25-LHR-151–249) travels almost collinearly (small angle of < 0.5°) with the excitation beam and is focused by a lens (*f* = 0.30 m), 5 cm before the sample, probing the induced phase shift in the sample. The intensity variation of the probe beam centre after passing through the sample was detected by a pinhole-laser line filter-photodetector assembly in the far field (>5 m from the sample). The laser line filter is used to prevent the excitation laser beam and ambient light from being detected by a fast photodetector (Femto, Model OE-300-SI-10-FST, 200 MHz bandwidth). A digital oscilloscope (Tektronix, Model DPO4102B) recorded the signal. The excitation beam was used to trigger the oscilloscope by using another photodetector (same as the probe sensor) at a repetition frequency of 10 Hz. The entire setup was aligned on precise actively damped optical tables to eliminate mechanical vibration of the sample. A heating unit and a temperature controller (Lakeshore, Model 340) were used to maintain the samples temperature at (298.15 ± 0.08) K. The excitation and probe beam radii were measured with a beam profile camera (Coherent, Model Lasercam HR). The laser energy was measured using a pyroelectric energy sensor (Thorlabs, Model ES120C). The experimental parameter, defined in the next section, *V* = 5.1. The uncertainties are smaller than 1% and correspond to the standard deviation of the mean over the course of experiments. A minimum of 15 measurements were performed for each sample and excitation energy with 512 transients averaged on each measurement. Figure 2a shows these averaged transients for samples with different thicknesses.

### PIL phase shift and signal

Considering an axially symmetric laser beam propagating along the *z*-axis of a semi-transparent material, the optical path is given by^55^

5 $$\phi ^i(r,t) = \mathop {\int }\limits_{\mathrm{path}} n^i(r,z,t)dz$$

where $n^i(r,z,t) = n_0 + {{{\mathrm{{\Delta}}}}}n^i$ and *i* = *w* refers to water and *i* = *g* to fused silica glass. The induced changes in the index ${{{\mathrm{{\Delta}}}}}n^i$ have contributions from temperature (${{{\mathrm{{\Delta}}}}}n_T^i$), pressure (${{{\mathrm{{\Delta}}}}}n_p^i$) and optical Kerr (${{{\mathrm{{\Delta}}}}}n_k^i$) effects. In the first approximation, these effects on the refractive index can be easily formulated as linearly dependent over the temperature, pressure and intensity range of interest by

6 $${{{\mathrm{{\Delta}}}}}n_j^i = \left( {\frac{{\partial n^i}}{{\partial x_j}}} \right)x_j^i(r,z,t)$$

*x*_*j*_ denotes the dependence of refractive index on the temperature $T^i(r,z,t)$, with the thermo-optic coefficient $\partial n^i/\partial T$, pressure $p^i(r,z,t)$, with the piezo-optic coefficient $\partial n^i/\partial p$, and intensity $I^i(r,z,t)$, with the non-linear refractive index $n_2^i = \partial n^i/\partial I$. These contributions add up to produce the phase shift to the probe beam as

7 $${{{\mathrm{{\Phi}}}}}\left( {r,z,t} \right) = \frac{{2\pi }}{{\lambda _{{{\mathrm{p}}}}}}\mathop {\sum}\nolimits_i {\phi ^i\left( {r,z,t} \right)}$$

where *λ*_p_ is the probe beam wavelength. Considering only the centre of the probe beam spot at the detector plane in the far-field region, and using Fresnel diffraction theory, the relative far-field intensity signal *S*(*t*) results in^54^

8 $$S\left( t \right) = \left| {{\int}_0^\infty {\frac{{2r}}{{w_{{{\mathrm{p}}}}^2}}{{{\mathrm{exp}}}}} \left[ { - \left( {1 + {{{\mathrm{i}}}}V} \right)\frac{{r^2}}{{w_{{{\mathrm{p}}}}^2}} - {{{\mathrm{i}}}}{\Phi}(r,z,t)} \right]{{{\mathrm{d}}}}r} \right|^2$$

where *V* is an experimental parameter and *w*_p_ the radius of the probe beam in the sample. The experimental parameters are listed in Fig. 2a. Equation (8) is evaluated numerically. The calculation of *S*(*t*) requires the determination of *T*^*i*^(*r*, *z*, *t*) and *p*^*i*^(*r*, *z*, *t*) fields considering all the effects of the radiation forces in the liquid and in the cuvette walls. The PIL signal shown in Fig. 2a, b is normalized as *S*(*t*)/*S*(0)−1.

### Pressure and temperature changes due to radiation forces

Finite element analysis has been used for the numerical calculations of pressure and temperature distributions using the software Comsol Multiphysics. The following equations are considered

9 $$\nabla ^2p(r,z,t) - \frac{1}{{v^2}}\partial _t^2p(r,z,t) = \nabla \cdot {{{\mathbf{f}}}} - \frac{{\beta A_{{{\mathrm{e}}}}}}{{c_P}}\partial _tI(r,z,t)$$

and

10 $$\rho _{{{\mathrm{m}}}}c_P\partial _tT(r,z,t) - k\nabla ^2T(r,z,t) = A_{{{\mathrm{e}}}}I(r,z,t)$$

where *p*(*r*, *z*, *t*) and *T*(*r*, *z*, *t*) are the pressure and temperature distributions, respectively, *v* is the sound velocity in the medium, *β* is the thermal expansion coefficient, *c*_*p*_ is the specific heat at constant pressure, *k* is the thermal conductivity, *A*_e_ is the optical absorption coefficient at the excitation wavelength, *I*(*r*, *z*, *t*) is the axially symmetric laser source intensity under pulsed excitation and **f** accounts for the body force densities inside the medium, which will be given by the electromagnetic force density in addition to the gravitational force density, i.e., **f** = **f**_**em**_ + *ρ*_m_**g**. Equation (9) is valid for pressure fields of small amplitude propagating in inviscid fluids. The radial component of **f**_em_ is given by

11 $$f_{{{{\mathrm{em}}}}}^{{{\mathrm{H}}}}\left( {r,t} \right) = - \frac{{8r}}{{cw_{{{\mathrm{e}}}}^2}}\frac{{(n^2 - 1)(n^2 + 2)}}{{3(n + 1)^2}}I\left( {r,t} \right)$$

for the Helmholtz formulation and

12 $$f_{{{{\mathrm{em}}}}}^{{{{\mathrm{MA}}}}}\left( {r,t} \right) = - \frac{{8r}}{{cw_{{{\mathrm{e}}}}^2}}\frac{{(n - 1)}}{{(n + 1)}}I\left( {r,t} \right)$$

for the Microscopic Ampère formulation.

The laser beam is linearly polarized in the fundamental Gaussian mode with nearly constant beam waist along the sample’s thicknesses. As the optical absorption along the sample length is very small, the intensity distribution is constant in the *z*-axis. These assumptions allow us to describe *I*(*r*, *z*, *t*) and **f**_em_ in terms of the cylindrical variable *r* and the time *t* only. As the gravitational force is constant, the pressure *p*(*r*, *z*, *t*) will then also be a function of *r* and *t* only.

The model was built in the 2D axisymmetric geometry. Pressure, temperature and intensity are calculated and the results are then used to generate the numerical simulations for the photo-induced lensing signal. The physical parameters of water and fused silica used in the simulations are presented in Supplementary Table S2.

### Equality of the force densities in Eqs. (1) and (3) for optical fields in non-magnetic media

To show that Eqs. (1) and (3) are indeed equal for optical fields in non-magnetic media, it is sufficient to focus on showing that the second term of Eq. (1) is equal to the first term of (3) as such other terms of these equations that are nonzero for non-magnetic media are identical. We start with the well-known relation between the polarization field and the electric field, given by

13 $${{{\mathbf{P}}}} = \varepsilon _0(\varepsilon _{{{\mathrm{r}}}} - 1){{{\mathbf{E}}}}$$

We assume that **P** is defined as the induced dipole moment density, i.e., ${{{\mathbf{P}}}} = N\langle {{{\mathbf{p}}}}\rangle$, where $\langle {{{\mathbf{p}}}}\rangle = \alpha {{{\mathbf{E}}}}$ is the average induced dipole moment, with polarizability *α*, and *N* is the number density of induced dipoles. Assuming that *m* is the mass associated to a volume of the material where a single dipole has been induced, one can write

14 $${{{\mathbf{P}}}} = \rho _{{{\mathrm{m}}}}\alpha {{{\mathbf{E}}}}/m$$

where $\rho _{{{\mathrm{m}}}} = mN$ is the mass density. Setting Eqs. (13) and (14) equal, we obtain $\varepsilon _0(\varepsilon _{{{\mathrm{r}}}} - 1) = \rho _{{{\mathrm{m}}}}\alpha /m$, from which we get

15 $$\varepsilon _{{{\mathrm{r}}}} = 1 + \frac{{\rho _{{{\mathrm{m}}}}\alpha }}{{\varepsilon _0m}}$$

Differentiating this equation with respect to the mass density, we obtain $\partial \varepsilon _{{{\mathrm{r}}}}/\partial \rho _{{{\mathrm{m}}}} = \alpha /(\varepsilon _0m)$. After substituting Eq. (15), the electrostriction coefficient becomes

16 $$\rho _{{{\mathrm{m}}}}\frac{{\partial \varepsilon _{{{\mathrm{r}}}}}}{{\partial \rho _{{{\mathrm{m}}}}}} = \frac{{\rho _{{{\mathrm{m}}}}\alpha }}{{\varepsilon _0m}} = \varepsilon _{{{\mathrm{r}}}} - 1$$

Consequently, the electrostriction term of the force density in Eq. (1) can be written as

17 $$\frac{1}{2}\varepsilon _0\nabla \left[ {\rho _{{{\mathrm{m}}}}\left( {\frac{{\partial \varepsilon _{{{\mathrm{r}}}}}}{{\partial \rho _{{{\mathrm{m}}}}}}} \right)_T|{{{\mathbf{E}}}}|^2} \right] = \frac{1}{2}\nabla ({{{\mathbf{P}}}} \cdot {{{\mathbf{E}}}})$$

The righthand side is identical to the first term of Eq. (3). Thus, the force densities in Eqs. (1) and (3) are equal.

The equality in Eq. (17) does not hold in the limit of static or quasi-static fields, in which case the fields of the induced bound surface charges contribute in a way described by the Clausius–Mossotti relation. This explains how the force density in Eq. (1) can explain both the present experimental results for an optical field and the results of the classic experiment by Hakim and Higham^37^ for a static field.

## Supplementary information

Supplementary Information

## Data Availability

The data that support the findings of this study are available from the corresponding author on reasonable request.
