# Supplementary material for: Unveiling bulk and surface radiation forces in a dielectric liquid
Source: Light Sci Appl. 2022 Apr 20;11:103. doi: 10.1038/s41377-022-00788-7 (PMC9021243; doi:10.1038/s41377-022-00788-7)
Supplement: Supplementary file 1 — Supplementary Information [file 41377_2022_788_MOESM1_ESM.pdf]

Supplementary Information for
Unveiling bulk and surface radiation forces in a dielectric liquid

N. G. C. Astrath,[1, *] G. A. S. Flizikowski,[1] B. Anghinoni,[1] L. C. Malacarne,[1] M. L. Baesso,[1] T. Požar,[2] M. Partanen,[3] I. Brevik,[4] D. Razansky,[5, 6] and S. E. Bialkowski[7]

[1]*Department of Physics, Universidade Estadual de Maringá, Maringá, PR 87020-900, Brazil*
[2]*Faculty of Mechanical Engineering, University of Ljubljana, Ljubljana 1000, Slovenia*
[3]*Photonics Group, Department of Electronics and Nanoengineering, Aalto University, 00076 Aalto, Finland*
[4]*Department of Energy and Process Engineering, Norwegian University of Science and Technology, Trondheim N-7491, Norway*
[5]*Institute for Biomedical Engineering and Institute of Pharmacology and Toxicology, Faculty of Medicine, University of Zurich, 8057 Zurich, Switzerland*
[6]*Institute for Biomedical Engineering, Department of Information Technology and Electrical Engineering, ETH Zurich, 8093 Zurich, Switzerland*
[7]*Department of Chemistry and Biochemistry, Utah State University, UT 84322-0300, USA*

I. MICROSCOPIC AMPÈRE FORMULATION

Maxwell's equations in most general form are written as

$$\epsilon_0 \boldsymbol{\nabla} \cdot \mathbf{E} = \rho \tag{1}$$

$$\boldsymbol{\nabla} \cdot \mathbf{B} = 0 \tag{2}$$

$$\boldsymbol{\nabla} \times \mathbf{E} + \frac{\partial \mathbf{B}}{\partial t} = 0 \tag{3}$$

$$\frac{1}{\mu_0} \boldsymbol{\nabla} \times \mathbf{B} - \varepsilon_0 \frac{\partial \mathbf{E}}{\partial t} = \mathbf{J} \tag{4}$$

where $\mathbf{E}$ is the electric field, $\mathbf{B}$ is the magnetic induction field and ε_0 and μ_0 are the free-space permittivity and permeability, respectively. The source terms are ρ, the charge density, and $\mathbf{J}$, the current density. They contemplate generically all the microscopic bound sources and all free sources inside the medium.

Initially, we want to obtain the electromagnetic force density $\mathbf{f}$ acting on dielectric media with no free charges or currents. These force densities will be implicitly analyzed at positions $\mathbf{r} = (x, y, z)$ where only the dipolar contributions of the sources are assumed to be relevant. Mathematically, the electric charge and current densities are given by[1]

$$\rho(\mathbf{r}, t) = -(\mathbf{p} \cdot \boldsymbol{\nabla}) \delta^3(\mathbf{r}) \tag{5}$$

and

$$\mathbf{J}(\mathbf{r}, t) = \dot{\mathbf{p}} \delta^3(\mathbf{r}) - (\mathbf{m} \times \boldsymbol{\nabla}) \delta^3(\mathbf{r}) \tag{6}$$

where $\mathbf{p}$ and $\mathbf{m}$ are the dipole's electric and magnetic moment, respectively, t is the time, $\boldsymbol{\nabla} = (\partial_x, \partial_y, \partial_z)$, $\delta^3(\mathbf{r})$ is the three dimensional Dirac delta function, given by $\delta^3(\mathbf{r}) = \delta(x)\delta(y)\delta(z)$, and $\dot{\mathbf{p}} = \mathrm{d}\mathbf{p}/\,\mathrm{d}t$. The force acting on the dielectric is given by the continuous version of the experimental Lorentz force law,

$$\mathbf{F} = \int (\rho \mathbf{E} + \mathbf{J} \times \mathbf{B}) \, \mathrm{d}^3\mathbf{r} \tag{7}$$

which yields

$$\mathbf{F} = \int \left(\left[-(\mathbf{p} \cdot \boldsymbol{\nabla}) \delta^3(\mathbf{r}) \right] \mathbf{E} + \left[\dot{\mathbf{p}} \delta^3(\mathbf{r}) \right] \times \mathbf{B} - \left[(\mathbf{m} \times \boldsymbol{\nabla}) \delta^3(\mathbf{r}) \right] \times \mathbf{B} \right) \mathrm{d}^3\mathbf{r} \tag{8}$$

Integrating the last equation and dividing the result by the dielectric volume, we obtain the force density in the so called Microscopic Ampère (MA) formulation

* e-mail: ngcastrath@uem.br

$$\mathbf{f}_{\mathrm{MA}} = (\mathbf{P}\cdot\boldsymbol{\nabla})\,\mathbf{E} + \dot{\mathbf{P}}\times\mathbf{B} + \mathbf{M}\times(\boldsymbol{\nabla}\times\mathbf{B}) + (\mathbf{M}\cdot\boldsymbol{\nabla})\,\mathbf{B} \tag{9}$$

where $\mathbf{P}$ and $\mathbf{M}$ are the polarization and magnetization fields, given by $\mathbf{p}$ and $\mathbf{m}$ divided by the dielectric volume, respectively.

Eq. (9) is compatible with a point dielectric dipole located at the origin, considering its electric charge and current densities as an incompressible fluid system (i.e., $\boldsymbol{\nabla}\cdot\mathbf{u} = 0$ and $\partial_t\rho + \boldsymbol{\nabla}\cdot\mathbf{J} = 0$, where $\mathbf{u}$ is the non-relativistic velocity of the dipole's center of mass). Explicitly, the time derivative term is

$$\dot{\mathbf{P}}\times\mathbf{B} = \frac{\partial\mathbf{P}}{\partial t}\times\mathbf{B} + (\mathbf{u}\cdot\boldsymbol{\nabla})\,\mathbf{P}\times\mathbf{B} \tag{10}$$

As we are interested in systems where the dipole's velocity is much smaller than the speed of light inside the medium, the last term can be safely neglected.

Using the vector property $\boldsymbol{\nabla}(\mathbf{U}\cdot\mathbf{V}) = (\mathbf{U}\cdot\boldsymbol{\nabla})\mathbf{V} + (\mathbf{V}\cdot\boldsymbol{\nabla})\mathbf{U} + \mathbf{U}\times(\boldsymbol{\nabla}\times\mathbf{V}) + \mathbf{V}\times(\boldsymbol{\nabla}\times\mathbf{U})$, we have

$$(\mathbf{P}\cdot\boldsymbol{\nabla})\,\mathbf{E} = \boldsymbol{\nabla}(\mathbf{P}\cdot\mathbf{E}) - (\mathbf{E}\cdot\boldsymbol{\nabla})\,\mathbf{P} - \mathbf{E}\times(\boldsymbol{\nabla}\times\mathbf{P}) - \mathbf{P}\times(\boldsymbol{\nabla}\times\mathbf{E}) \tag{11}$$

and

$$\mathbf{M}\times(\boldsymbol{\nabla}\times\mathbf{B}) + (\mathbf{M}\cdot\boldsymbol{\nabla})\,\mathbf{B} = \boldsymbol{\nabla}(\mathbf{M}\cdot\mathbf{B}) - \mathbf{B}\times(\boldsymbol{\nabla}\times\mathbf{M}) - (\mathbf{B}\cdot\boldsymbol{\nabla})\,\mathbf{M} \tag{12}$$

For linear isotropic media, the medium responses are given by $\mathbf{P} = \varepsilon_0\chi_{\mathrm{e}}\mathbf{E}$ and $\mathbf{M} = \chi_{\mathrm{m}}\mathbf{H}$, where χ_{e} and χ_{m} are the electric and magnetic susceptibilities, respectively, and $\mathbf{H} = \mathbf{B}/\mu_0 - \mathbf{M}$ is the magnetic field. Working on Eq. (11), we have

$$(\mathbf{P}\cdot\boldsymbol{\nabla})\,\mathbf{E} = \varepsilon_0\boldsymbol{\nabla}\left(\chi_{\mathrm{e}}|\mathbf{E}|^2\right) - \varepsilon_0(\mathbf{E}\cdot\boldsymbol{\nabla})(\chi_{\mathrm{e}}\mathbf{E}) - \varepsilon_0\mathbf{E}\times(\boldsymbol{\nabla}\times(\chi_{\mathrm{e}}\mathbf{E})) - \varepsilon_0\chi_{\mathrm{e}}\mathbf{E}\times(\boldsymbol{\nabla}\times\mathbf{E}) \tag{13}$$

Analogously, for Eq. (12),

$$\mathbf{M}\times(\boldsymbol{\nabla}\times\mathbf{B}) + (\mathbf{M}\cdot\boldsymbol{\nabla})\,\mathbf{B} = \boldsymbol{\nabla}(\chi_{\mathrm{m}}\mathbf{H}\cdot\mu\mathbf{H}) - \mu\mathbf{H}\times(\boldsymbol{\nabla}\times\chi_{\mathrm{m}}\mathbf{H}) - (\mu\mathbf{H}\cdot\boldsymbol{\nabla})(\chi_{\mathrm{m}}\mathbf{H}) \tag{14}$$

where $\mu = \mu_0(\chi_{\mathrm{m}} + 1)$ is the medium's permeability.

By applying elementary vector properties to the last two equations and adding the hidden momentum contribution[6] as $-\partial_t(\mathbf{M}\times\mathbf{E})/c^2$, Eq. (9) can be rewritten as

$$\mathbf{f}_{\mathrm{MA}} = \frac{\varepsilon_0(\varepsilon_{\mathrm{r}}-1)}{2}\boldsymbol{\nabla}|\mathbf{E}|^2 + (\mu_{\mathrm{r}}-1)|\mathbf{H}|^2\boldsymbol{\nabla}\mu + \frac{\mu(\mu_{\mathrm{r}}-1)}{2}\boldsymbol{\nabla}|\mathbf{H}|^2 + \frac{n^2-1}{c^2}\frac{\partial}{\partial t}(\mathbf{E}\times\mathbf{H}) \tag{15}$$

where we used $\chi_{\mathrm{e}} = \varepsilon_{\mathrm{r}} - 1$ and $\chi_{\mathrm{m}} = \mu_{\mathrm{r}} - 1$. Here, $n = \sqrt{\varepsilon_{\mathrm{r}}\mu_{\mathrm{r}}}$ is the refractive index, $\varepsilon_{\mathrm{r}} = \varepsilon/\varepsilon_0$ is the relative permittivity and $\mu_{\mathrm{r}} = \mu/\mu_0$ is the relative permeability, which can all depend on position, but not on time. A simple rearrangement of the gradients as products yields then

$$\mathbf{f}_{\mathrm{MA}} = \frac{1}{2}\boldsymbol{\nabla}(\mathbf{P}\cdot\mathbf{E}) + \frac{1}{2}\boldsymbol{\nabla}(\mathbf{M}\cdot\mathbf{B}) - \frac{1}{2}|\mathbf{E}|^2\boldsymbol{\nabla}\varepsilon - \frac{1}{2}|\mathbf{H}|^2\boldsymbol{\nabla}\mu + \frac{n^2-1}{c^2}\frac{\partial}{\partial t}(\mathbf{E}\times\mathbf{H}) \tag{16}$$

If the excitation is harmonic and the medium is non-magnetic ($\mu_{\mathrm{r}} = 1$), the time-average ($\langle\ \ \rangle$) over an optical cycle yields

$$\langle\mathbf{f}_{\mathrm{MA}}\rangle = \frac{\varepsilon_0(n^2-1)}{4}\boldsymbol{\nabla}|\mathbf{E}|^2 \tag{17}$$

For a gaussian beam, the volume force generated is a compression in the radial direction, independent of the polar angle. This is qualitatively identical to the known phenomenological electrostriction effect, which, according to Helmholtz formulation[2], is given by

$$\langle\mathbf{f}_{\mathrm{H}}\rangle = \varepsilon_0\frac{\gamma}{4}\boldsymbol{\nabla}|\mathbf{E}|^2 \tag{18}$$

where γ is the electrostriction coefficient, $\gamma = \rho_{\mathrm{m}}\left(\frac{\partial\varepsilon_{\mathrm{r}}}{\partial\rho_{\mathrm{m}}}\right)_T$, with ρ_{m} being the mass density and T the temperature. The relative magnitude of the force densities is

$$\frac{\langle f_{\mathrm{MA}}\rangle}{\langle f_{\mathrm{H}}\rangle} = \frac{n^2-1}{\gamma} \tag{19}$$

If we apply the Clausius-Mossotti relation, we have $\gamma = (\varepsilon_{\mathrm{r}}-1)(\varepsilon_{\mathrm{r}}+2)/3$. Then, for water, with $n = 1.33$,

$$\frac{\langle f_{\mathrm{MA}}\rangle}{\langle f_{\mathrm{H}}\rangle} \approx 0.796 \tag{20}$$

The difference of the force density in the Microscopic Ampère formulation is compared to the main existing formulations, as given in Table S1.

Table S1. Comparing the Microscopic Ampère force density with other formulations.

**Formulation**	**Difference in force density**
Minkowski	$\mathbf{f}_{\rm M}-\mathbf{f}_{\rm MA}=-\frac{1}{2}\boldsymbol{\nabla}(\mathbf{P}\cdot\mathbf{E})-\frac{1}{2}\boldsymbol{\nabla}(\mathbf{M}\cdot\mathbf{B})-\frac{n^2-1}{c^2}\frac{\partial}{\partial t}(\mathbf{E}\times\mathbf{H})$
Abraham	$\mathbf{f}_{\rm Ab}-\mathbf{f}_{\rm MA}=-\frac{1}{2}\boldsymbol{\nabla}(\mathbf{P}\cdot\mathbf{E})-\frac{1}{2}\boldsymbol{\nabla}(\mathbf{M}\cdot\mathbf{B})$
Conventional Ampère	$\mathbf{f}_{\rm A}-\mathbf{f}_{\rm MA}=-\overleftrightarrow{\boldsymbol{\nabla}}\cdot(\mathbf{P}\otimes\mathbf{E})+\overleftrightarrow{\boldsymbol{\nabla}}\cdot(\mathbf{B}\otimes\mathbf{M})-\boldsymbol{\nabla}(\mathbf{M}\cdot\mathbf{B})$
Einstein-Laub	$\mathbf{f}_{\rm EL}-\mathbf{f}_{\rm MA}=-\frac{\mu_0}{2}\boldsymbol{\nabla}\lvert\mathbf{M}\rvert^2$
Chu	$\mathbf{f}_{\rm C}-\mathbf{f}_{\rm MA}=-\overleftrightarrow{\boldsymbol{\nabla}}\cdot(\mathbf{P}\otimes\mathbf{E})-\mu_0\overleftrightarrow{\boldsymbol{\nabla}}\cdot(\mathbf{H}\otimes\mathbf{M})-\frac{\mu_0}{2}\boldsymbol{\nabla}\lvert\mathbf{M}\rvert^2$

II. RESIDUALS

Calculated residuals of PIL and PMM transients are shown below in Figs. S1 and S2, respectively.

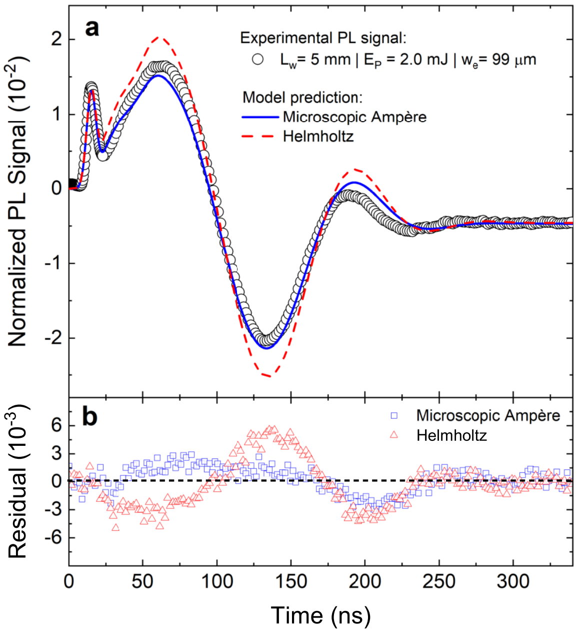

Figure S1. **Time-dependent photo-induced lensing (PIL) transients**. **a** PIL signal under pulsed laser excitation at 532 nm for $L_w = 5$ mm. Open symbols are experimental data and continuous lines represent the numerical calculations using $S(t)$; confidence level of 95%. The uncertainties in a are smaller than 1% and correspond to the standard deviation of the mean over all the experiments (see Methods). **b**, Calculated residuals.

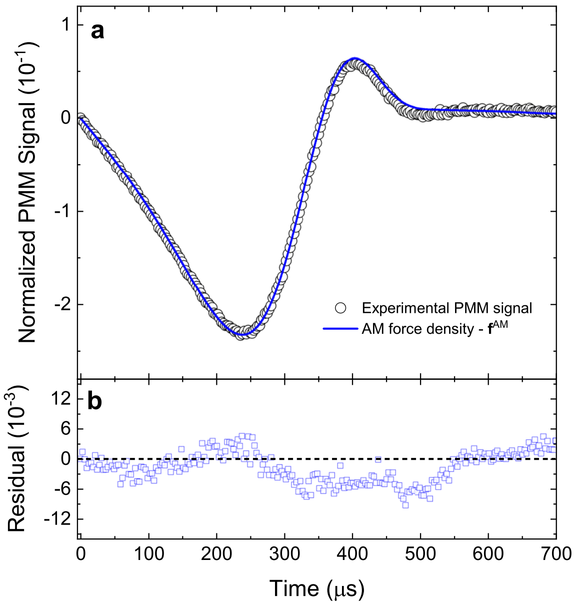

Figure S2. **Photomechanical mirror (PMM) measurements**. **a**, PMM signal under pulsed laser excitation at 532 nm. Open symbols are experimental data and continuous line represents the numerical calculations using Eq. (5). Dashed line shows the Helmholtz model prediction. **b**, Calculated residuals.

III. PHYSICAL PROPERTIES USED IN THE SIMULATIONS

The physical parameters of water and fused silica used in the simulations are presented in Table S2.

Table S2. Supplementary Table: Physical properties used in the simulations[3,4].

Parameter	Units	Water	Fused silica
$\partial n/\partial p$	10^{-10} Pa^{-1}	1.4[5]	0.031
$\partial n/\partial T$	10^{-6} K^{-1}	−96	−9.6
v	m s^{-1}	1481	5800
n_2	10^{-20} m^2 W^{-1}	1.0	1.7
A_e	m^{-1}	0.045[7]	0.005
β	10^{-6} K^{-1}	261	0.55
c_P	J kg^{-1} K^{-1}	4182	703
k	W m^{-1} K^{-1}	265	507
ρ_m	kg m^{-3}	998	2203

[1] Griffiths, D. J. & Hnizdo, V. What's the use of bound charge?. Preprint at http://arxiv.org/abs/1506.02590 (2015).
[2] Landau, L. D. & Lifshitz, E. M. Electrodynamics of continuous media. (Oxford: Pergamon Press, 1984).
[3] Lide, D. R. CRC Handbook of chemistry and physics. 88th edn. (Cleveland: CRC Press, 1977).
[4] Capeloto, O. A. *et al.* Nanosecond pressure transient detection of laser-induced thermal lens. *Applied Optics* **59**, 3682-3685 (2020).
[5] Cho, C. H. & Urquidi, J. Mixture model description of the T-, P dependence of the refractive index of water. *The Journal of Chemical Physics* **114**, 3157-3162 (2001).
[6] Griffiths, D. J. Resource letter EM-1: Electromagnetic momentum. *American Journal of Physics* **80**, 7-18 (2012).
[7] Sogandares, F. M. & Fry, E. S. Absorption spectrum (340-640 nm) of pure water. I. Photothermal measurements. *Applied Optics* **36**, 8699-8709 (1997).
